# The Longitudinal and Vertical Integration of Health Systems Science in an Undergraduate Medical Education Program: A Means to Support “Curricular Elasticity"

**DOI:** 10.7759/cureus.38591

**Published:** 2023-05-05

**Authors:** Dimitrios Papanagnou, Deborah Ziring

**Affiliations:** 1 Department of Emergency Medicine, Thomas Jefferson University, Philadelphia, USA; 2 Department of Medicine, Thomas Jefferson University, Philadelphia, USA

**Keywords:** curricular innovation, curriculum planning, curriculum design, undergraduate medical education, health systems science

## Abstract

Although health systems science (HSS) has become increasingly included as requisite curricular content in undergraduate medical education (UME), educators have many implementation options for integrating HSS content into medical school training. Learning from medical schools’ authentic experiences and lessons learned for the successful and sustainable implementation of HSS would be valuable. We share our experience with the longitudinal and vertical integration of HSS at the Sidney Kimmel Medical College (SKMC) at Thomas Jefferson University in Philadelphia over the past six years. We posit that our approach to curricular design has afforded us the “curricular elasticity” needed to keep our educational program current and flexible in the rapidly changing healthcare and geopolitical landscape.

## Editorial

Although health systems science (HSS) has become increasingly included as requisite curricular content in undergraduate medical education (UME) [1], educators have many implementation options for integrating HSS content into medical school training. Learning from medical schools’ authentic experiences and lessons learned for the successful and sustainable implementation of HSS would be valuable. We share our experience with the longitudinal and vertical integration of HSS at the Sidney Kimmel Medical College (SKMC) at Thomas Jefferson University in Philadelphia over the past six years. We posit that our approach to curricular design has afforded us the “curricular elasticity” needed to keep our educational program current and flexible in the rapidly changing healthcare and geopolitical landscape. 

Supported by the American Medical Association, medical schools have increasingly integrated HSS into their respective formal curricula to better prepare trainees to thrive in today’s healthcare system. Described as the third educational pillar that complements the basic and clinical sciences, HSS provides a framework of competencies related to value-based care, population health, interprofessional collaboration, health system improvement, and systems thinking [2]. Through coursework in HSS, students have the opportunity to learn how healthcare is delivered, how teams of healthcare professionals can work together to deliver care, and how the health system can better support patient care and care delivery. As we describe below, we intentionally incorporated HSS as a curricular thread in our medical education program.

SKMC underwent a significant curricular transformation (JeffMD) in 2017, transitioning from a 2+2 paradigm (i.e., two years of pre-clinical coursework in the basic sciences, followed by two years of clinical clerkships in the clinical environment) to a three-phase curriculum integrating basic science, clinical science, and HSS throughout all four years [3]. Rather than being confined to a single course, HSS was integrated as a curricular thread spanning all three phases. Students are introduced to HSS in Phase 1 during our small-group case-based learning (CBL) component. Each case includes HSS-specific learning objectives that align with our organ-system curriculum. In addition to HSS lectures and patient panels in Phase 1, where patient educators are invited to share their lived experiences with the health system, students are immersed in practical clinical experience sessions introducing them to HSS-related domains. During their first year of medical school, they partner with community health workers at outpatient sites for exposure to the challenges patients face and the impact of social and structural determinants of health on healthcare delivery [3].

Exposure to HSS continues in Phases 2 and 3 through clinical simulations in the clinical clerkships, summative objective structured clinical examinations (OSCEs), and skills-specific workshops (i.e., communicating diagnostic uncertainty to patients during transitions in care). Clerkship and course directors are expected to design educational experiences with learning objectives that link to HSS-specific content domains. Phase 2 and Phase 3 Directors record all educational experiences from their respective thread and track HSS learning objectives over the course of years three and four of training. Little did we know that our design for HSS would afford our medical school with the “curricular elasticity” to address several geopolitical crises over recent years.

Essentially, HSS at SKMC has provided the essential curricular space to address critical conversations with our students in real time. These included systemic racism, the COVID-19 pandemic, gender identity, disability, reproductive health and firearm control, along with other political and social determinants of health. Because HSS is represented throughout students’ training at SKMC, we were able to seamlessly incorporate current events quickly. This ability enabled us to fit these crucial topics into the curriculum for each medical school cohort concurrently. While other schools may have addressed these issues extracurricularly, or with only one or two classes of students at a specific point in time, our structural design allowed us to address these conversations in the formal curriculum. Working with students, we rapidly co-produced curricular content and then delivered it within the HSS thread.

For example, following Dobbs v. Jackson Women’s Health Organization (2022), in which the United States Supreme Court overturned Roe v. Wade (1973), we leveraged one of our HSS patient panels to address this. During the Reproductive Block in Phase 1, we modified a panel session to: a) discuss the impact of legislation on patient autonomy; b) recognize the impact of legislation on healthcare providers; and c) highlight the impact of legislation on training future clinicians. Similarly, SKMC’s Race and Racism in Medicine curriculum was linked to specific HSS curricular learning objectives immediately following the death of George Floyd (2020) [4]. The longitudinal integration of HSS allows our students to revisit conversations surrounding systemic racism throughout their training. The dynamic events during the COVID-19 pandemic and the impact of social determinants of health on patient outcomes are other key examples [5].

Reflecting on lessons learned, we have come to appreciate the “elasticity” an HSS curriculum affords a medical school to address high-stakes conversations in real time. Medical students require this training to prepare them for the dynamic nature of HSS, including the complexity and uncertainty of clinical practice. Our structural design of longitudinal, vertical integration of HSS has allowed us to successfully fulfill this need. We have also learned that a single curricular experience (i.e., patient panel, lecture, or CBL case) is often insufficient. In response to student feedback, we now include a one-hour optional debrief immediately following sessions on gender identity and the history of racism in medicine.

In summary, HSS, when longitudinally integrated, offers the ideal framework to flexibly address unanticipated content on significant current events impacting the healthcare landscape. It has liberated us from relying on low-impact standalone sessions and enabled rapid response. We believe the vertical integration of HSS is a vehicle to ensure the elasticity our medical school curriculum requires to continuously adapt in a complex healthcare landscape heavily influenced by geopolitical forces. We intend to continue this approach at SKMC, as well as the invaluable engagement of HSS student liaisons to co-produce curricular content.
